# Impact of a Face-To-Face Versus Smartphone App Versus Combined Breastfeeding Intervention Targeting Fathers: Randomized Controlled Trial

**DOI:** 10.2196/24579

**Published:** 2021-04-12

**Authors:** Jane Anne Scott, Sharyn K Burns, Yvonne L Hauck, Roslyn C Giglia, Anita M Jorgensen, Becky Kate White, Annegret Martin, Suzanne Robinson, Satvinder S Dhaliwal, Colin W Binns, Bruce R Maycock

**Affiliations:** 1 School of Population Health Curtin University Perth Australia; 2 School of Nursing, Midwifery and Paramedicine Curtin University Perth Australia; 3 Curtin Health Innovation Research Institute Curtin University Perth Australia; 4 Duke-NUS Medical School National University of Singapore Singapore Singapore; 5 KK Women's and Children's Hospital Singapore Singapore

**Keywords:** breastfeeding, fathers, peer support, mHealth, smartphone app, infants, social support, feeding, smartphone

## Abstract

**Background:**

Despite the recognized health and economic benefits of exclusive breastfeeding, few Australian infants are exclusively breastfed beyond 5 months of age. Social support for breastfeeding, in particular the support of an infant’s father, has been identified as a crucial element for successful breastfeeding.

**Objective:**

The objective of this study was to determine the effectiveness of various father-focused breastfeeding interventions in terms of key infant feeding outcomes.

**Methods:**

The study was a 4-arm, factorial, randomized controlled trial conducted in Perth, Australia. The trial arms included a control group and 3 interventions, consisting of a face-to-face father-focused antenatal breastfeeding class facilitated by a male peer facilitator; Milk Man, a breastfeeding smartphone app designed specifically for fathers; and a combination of both interventions. Expecting couples were recruited from hospital-based antenatal classes and block randomized to 1 of the 4 arms. Each partner completed surveys at recruitment and at 6 weeks and 26 weeks postpartum. Primary outcomes were duration of exclusive and any breastfeeding. Secondary outcomes included age of introduction of formula and complementary foods, maternal breastfeeding self-efficacy, and partner postpartum support.

**Results:**

A total of 1426 couples were recruited from public (443/1426, 31.1%) and private (983/1426, 68.9%) hospitals. Of these, 76.6% (1092/1426) of fathers completed the baseline questionnaire, 58.6% (836/1426) completed the 6-week follow-up questionnaire, and 49.2% (702/1426) completed the 26-week follow-up questionnaire. The average age of fathers who completed the baseline questionnaire was 33.6 (SD 5.2) years; the majority were born in Australia (76.4%) and had attended university (61.8%). There were no significant differences between the control and any of the intervention groups in any of the infant feeding outcomes or level of breastfeeding self-efficacy and postpartum partner support reported by mothers.

**Conclusions:**

This study did not demonstrate that any intervention was superior to another or that any intervention was inferior to the standard care delivered in routine antenatal classes. Further studies are needed to test the effectiveness of these interventions in more socioeconomically diverse populations that are likely to benefit most from additional partner supports.

**Trial Registration:**

Australian New Zealand Clinical Trials Registry ACTRN12614000605695; https://www.anzctr.org.au/Trial/Registration/TrialReview.aspx?ACTRN=12614000605695

**International Registered Report Identifier (IRRID):**

RR2-10.1186/s12884-015-0601-5

## Introduction

### Breastfeeding and Fathers

Breastfeeding is known to have short- and long-term health benefits for both infants [1,2] and mothers [3]. Despite the well-substantiated health [4] and economic [5,6] benefits of breastfeeding and high breastfeeding initiation rates (95%) [7], only 15% of Australian infants are exclusively breastfed beyond 5 months, and less than 6 out of every 10 still receive any breast milk at 6 months of age [7]. These statistics have remained relatively stagnant for the last 25 years or so [8,9], and new and innovative ways of increasing the duration and exclusivity of breastfeeding are needed to ensure that most Australian infants (and their mothers) receive the maximum and continued benefits of breastfeeding.

Social support for breastfeeding [10,11] and in particular support of the babies’ fathers have been identified as crucial elements for successful breastfeeding. While family structure varies, research to date has focused on male partners, as does this paper. A woman’s partner can act as a strong enabler or barrier to breastfeeding. There is sound empirical evidence that women who perceive their partners to be supportive of breastfeeding are more likely to initiate breastfeeding and to breastfeed for longer than women who perceive their partners to favor formula feeding or to be ambivalent as to how they feed their infant [12-16]. These findings are supported by a rapidly growing body of qualitative evidence that breastfeeding women value and benefit from the emotional and practical support of their partner [17-20].

While fathers typically describe breastfeeding as being normal and natural and want to be supportive of their breastfeeding partners, they are often poorly informed about the importance of breastfeeding and its superiority over formula feeding [21]. In addition, they can hold negative attitudes regarding breastfeeding including feeling left out, fear of not bonding with their infant, and of losing time with, and the attention of, their partner [13]. Fathers want to be involved in the breastfeeding decision-making process [20,22], and new fathers want practical advice on how they can support their partner as well as strategies for problem solving common breastfeeding difficulties that their partner may encounter [23].

However, while expecting fathers are encouraged to and frequently do attend antenatal classes with their partners, these classes are generally directed at the mothers and led by female health professionals, with men perceiving that they pay limited attention to their role and information and support needs [20]. Furthermore, work commitments may limit a father’s involvement in his partner’s pregnancy care and the number of antenatal classes and appointments that he can attend [24]. Information and support, therefore, need to be targeted toward men in a way that is accessible, flexible, and appropriate [24].

The authors [25], and others [26-28], have employed father-focused breastfeeding education classes led by male peer facilitators to provide expecting fathers with practical and nonauthoritative information and advice around providing breastfeeding support for their partners. Fathers participating in classes may feel less embarrassed or intimidated in expressing their concerns and asking questions of a peer father compared with a female health professional [29]. Face-to-face programs of this kind have enhanced the knowledge and ability of expecting fathers to support their breastfeeding partner [26,29] and have resulted in increased rates of breastfeeding initiation [28,29] and modest increases in breastfeeding duration [25]. Peer support programs of this kind, however, while valued by fathers and health professionals, are labor intensive and difficult and expensive to sustain. Digital technologies, with their wide geographic and demographic reach, provide a potentially cost-effective and sustainable means of reaching large numbers of individuals directly with health information, support, and interventions [30].

### Engaging With Fathers via Digital Technology

Mobile health (mHealth) interventions employing digital technologies provide a rapidly evolving means of engaging fathers and providing them with information and support to address their needs related to both breastfeeding and transitioning to fatherhood. Expecting and new parents, both mothers and fathers, have traditionally accessed the internet for information on pregnancy and early parenting [31,32], but increasingly they are accessing digital media information sources such as apps and social media platforms for this information [31,33].

The perinatal period provides a window of opportunity for connecting with fathers at a time when they are experiencing change, highly motivated, and looking for support [14]. Increasingly, men are seeking information and skills to enhance parenting and infant care (including breastfeeding), support and improve their relationship with their partner, and manage stress during this period [32]. They are accustomed to easy and immediate access to information using digital technologies and want better access to information than that offered by health professionals [33]. mHealth interventions can provide the user with readily accessible information despite geographical distance or time constraints, and the immediacy offered by digital technologies provides users with information when it is most needed [33]. Peer support can be provided through app-based online forums [34] and can assist the transition to fatherhood by providing fathers with the opportunity to share information and experiences, provide mutual support, and know they are not alone with their concerns [34,35]. The aim of this study was to implement and evaluate the effectiveness of 2 father-focused breastfeeding interventions, a face-to-face father-focused antenatal breastfeeding class and a breastfeeding smartphone app designed specifically for fathers, individually and in combination.

## Methods

The Parent Infant Feeding Initiative (PIFI) was a 4-arm, factorial, randomized controlled trial (RCT) conducted in Perth, Australia, and the study protocol has been described previously in detail [36].

### Participants and Recruitment

Participants were expecting couples recruited directly by members of the research team from 261 evening and weekend antenatal classes conducted between August 2015 and December 2016 at one public tertiary, 2 public regional, and 3 private hospitals providing maternity services to the majority of the Perth metropolitan area, with approximately 50% of metropolitan deliveries occurring in the private hospitals [37]. Only 2 smaller regional public hospitals were not included as recruitment sites for logistical reasons, due to the irregular scheduling of their antenatal classes.

Inclusion criteria included ownership by the father of a smartphone (iOS or Android), internet access, residence within Perth, both partners intending to participate in the rearing of their child, and having sufficient English language skills to engage with the intervention. Couples were excluded if the mother had an existing medical condition likely to inhibit the initiation of breastfeeding or exclusive breastfeeding, was expecting a multiple birth, or if they were a same sex couple.

### Interventions

The trial arms included a control group and 3 interventions consisting of (1) a face-to-face father-focused antenatal breastfeeding class (FFABC) facilitated by a male peer, (2) Milk Man, a breastfeeding smartphone app designed specifically for fathers, and (3) a combination of both interventions. Development of the individual interventions was informed by the social cognitive theory [38], which facilitated understanding of the potential interaction between overestimation of new parents’ capacity to cope and underestimation of potential problems.

All participants received a congratulatory card from the project on the birth of their baby. During the course of the study, couples in all groups may have accessed professional and community-based breastfeeding support services such as a lactation consultant, local breastfeeding support groups, or the Australian Breastfeeding Association’s website or 24-hour helpline. Fathers participating in the FFBAC were provided with a leaflet with contact numbers of relevant support services and encouraged to use these if needed. Similarly, the Milk Man app contained links to these same services and others that participants could access directly from within the app.

#### Father-Focused Antenatal Breastfeeding Class Group

The primary purpose of the FFABC was to identify and discuss ways that fathers can encourage and support their partners with breastfeeding. The format and content of the FFABC was based on a “dads only” breastfeeding class trialed in the Fathers Infant Feeding Initiative (FIFI) [25]. Details of the FFABC and its process evaluation have been reported previously [39].

Briefly, the FFABC was a single class that ran for approximately 45 minutes and was conducted at the time of the hospital-based couples’ antenatal class, replacing for fathers the usual breastfeeding component of that class with the father-focused class. The FFABC was led by a trained peer facilitator who was the father of at least one child aged younger than 3 years who had been breastfed for at least 3 months. The class explored issues identified in the literature [40-42] and confirmed in our earlier intervention [43] as being important to new fathers, including what it means to be a new father, the importance of breastfeeding, barriers and facilitators of breastfeeding, and anticipatory problem-solving strategies for addressing common breastfeeding problems.

#### Milk Man Smartphone App Group

The development of the Milk Man app, available for Android and iPhone (iOS, Apple Inc) operating systems, has been described in greater detail elsewhere [44]. Briefly, the app used gamification, social connectivity in the form of a conversation forum, and twice-weekly push notifications linking to polls and conversation starters to engage fathers with breastfeeding information contained within an information library. In addition to containing information on all of the topics introduced in the FFABC, the library contained additional breastfeeding and parenting information and links to external websites.

#### Combination Group

Fathers in the combination group had access to the Milk Man app from recruitment until 6 months postpartum and also attended the FFABC in place of the breastfeeding component of the hospital-based couples’ antenatal class.

Following randomization, participants in the Milk Man app and combination intervention groups were provided with instructions and an ID code for downloading the app. Milk Man app use was not prescribed and fathers had access to the app from recruitment at approximately 32 weeks’ gestation to 6 months postpartum, and app library content was unchanged for the duration of the study.

#### Control Group

Fathers in the control group received the usual care and attended the breastfeeding component of the hospital-based couples’ antenatal class.

### Randomization

To ensure close balance of participant numbers in each arm at any time during the trial, we used a block RCT to form the assignment list for the 4 study arms. Specifically, we used a computer-based random sequence generator to create random permuted blocks of 8 and an equal allocation ratio for each recruiting hospital, and then randomly assigned classes (of participants) within each block into one of the 4 study arms during the course of the 18 months of recruitment. This randomization process resulted in hospitals having roughly equivalent proportions of participants in each study arm (χ^2^_15_=22.8, *P*=.09). In view of this block randomization process, no effect of clustering was considered in our analysis.

Participants were blinded to the study arm allocation until after they had consented to participate. However, as some FFABCs were conducted on the same day as participants were recruited, it was necessary for members of the PIFI study team to be aware of the group allocation in order to organize for the peer facilitator to deliver the class. Care was taken by recruiting staff, through the use of a standardized slide presentation and recruitment script, to avoid inadvertently alerting potential participants to the study arm that their antenatal class had been allocated to, thereby influencing their decision to participate.

### Collection of Data

Each partner self-completed a printed baseline questionnaire collected at the time of recruitment or returned in a return-paid envelope. Follow-up questionnaires were completed at 6 weeks and 26 weeks postpartum. Each partner was sent an email with a personalized link to an online questionnaire, developed using Qualtrics software (Qualtrics). Three reminder emails were sent, followed by a final reminder by telephone, at which time participants had the option of completing the questionnaire by telephone survey.

From 36 weeks’ gestational age, fathers were sent a short message service (SMS) text asking if their baby had been born, and if so, the baby’s date of birth and sex. These messages stopped once notification of the baby’s birth was made, or at 42 weeks’ gestational age if fathers failed to respond before this time. In addition, mothers were sent a short 3-item survey, developed using Qualtrics software, at 12 weeks and 18 weeks postpartum via SMS text, with 3 reminder SMS texts, to determine if they had stopped breastfeeding and/or introduced formula or complementary (solid and semisolid) foods. A yes response to each of these questions generated a second question that requested mothers provide the age of their child in weeks when the relevant event occurred.

### Outcome Measurements

The primary outcomes were duration of exclusive and any breastfeeding. Secondary outcomes included age of introduction of formula, age of introduction of complementary foods, maternal breastfeeding self-efficacy, and partner postpartum support. Breastfeeding definitions were those used by the World Health Organization, and an infant was exclusively breastfed if they had received nothing but breastmilk (excluding oral rehydration solution or vitamins, minerals, or medicines given as drops or syrups) [45].

Infant feeding outcome measurements were derived from questions asked of both parents at 6 weeks and 26 weeks postpartum and of mothers at 12 weeks and 18 weeks postpartum via SMS text that related to current feeding method; age at which breastfeeding was stopped; and when formula, water, other beverages, or complementary foods were first started. Where outcome data were available from both parents, data collected from the mother were used on the assumption that these would be the more accurate and reliable. However, where data were only provided by the father, these data were used. In the event that neither parent completed the 26-week questionnaire, and to allow for survival analysis [46], the last available data from the 6-week follow-up questionnaire or the 12-week and 18-week SMS text surveys were used and right censored if necessary.

The 6 weeks postpartum follow-up questionnaire completed by mothers included 2 validated and widely used self-report instruments. The 14-item short form Breastfeeding Self-Efficacy Scale (BSES-SF) [47] assesses breastfeeding confidence. Scores can range from 14 to 70, with higher scores indicating higher levels of breastfeeding self-confidence. The 25-item Postpartum Partner Support Scale (PPSS) assesses functional elements of partner support, being appraisal/emotional, informational, and instrumental support. Scores can range from 25 to 100, with higher scores indicating higher levels of postpartum partner support [48].

### Statistical Analysis

Sample size was based on the proportion of women breastfeeding at 26 weeks. It was assumed that at 26 weeks, there would be at least a 10% difference in the proportion of women breastfeeding between any 2 of the groups. A sample size of 300 fathers was required in each of the 3 intervention groups and control group to be able to detect the difference at 80% power and 5% level of significance, using a log-rank survival test. Assuming a loss to follow-up of 25% in each group, 400 participants were to be recruited into each group.

Data were analyzed using the SPSS Statistics for Windows version 27 (IBM Corp). Multiple imputations of missing data were performed using fully conditional specification with iterative Markov chain Monte Carlo method. The imputations were performed for the 4 arms (ie, control, FFBAC, Milk Man, and combination) separately with specified value contrarians to ensure the accuracy of the imputed results. All imputations used 10 iterations to produce 100 imputed datasets (with 1000 case and 100 draws).

Binary logistic regression was conducted to estimate the odds ratio and 95% confidence interval of exclusive and any breastfeeding at 6 weeks and 26 weeks for the intervention groups versus the control group. Survival analysis using the Cox proportional hazard model was conducted to estimate the hazard ratio and 95% confidence interval in the intervention groups versus the control group for stopping exclusive and any breastfeeding and introducing formula or complementary foods before 26 weeks. The general linear model was used to compare the level of maternal breastfeeding self-efficacy (BSES-SF) and postpartum partner support (PPSS) reported by mothers. Results are presented as the mean and 95% confidence interval of the BSES-SF and PPSS scores, along with the regression coefficient, standard error, and *P* value obtained from the regression analyses. Results for all statistical tests are presented for the original analyses, which included those participants with complete data and the pooled analyses that used the imputed datasets, and *P*<.05 was considered to be statistically significant.

Intention-to-treat analysis was conducted according to the arm of the study that fathers were randomized to at recruitment. Per-protocol analysis was conducted on all control group fathers; those fathers randomized to the FFABC who had attended the class; those randomized to the Milk Man app group who had downloaded the app; and those randomized to the combination group who had attended the FFABC and downloaded the app.

### Ethics Approval and Consent to Participate

PIFI was approved by the Curtin University human research ethics committee (HR 82/2014; May 14, 2014) and the human research ethics committees responsible for the public (SCGG HREC No. 2014-111, Sept 18, 2014; SMHS HREC Reference S/15/25, Aug 27, 2015; WNHS HREC No. 2016037EW, May 4, 2016) and private (SJGHC Reference 777, April 8, 2015) hospital sites. The study was registered with the Australian New Zealand Clinical Trials Registry [ACTRN12614000605695]. Members of the research team attended each antenatal class and provided a verbal and written description of the study. Participation was voluntary, and all participants provided signed informed consent.

## Results

### Participants and Retention

In total, 1426 couples were recruited from public (443/1426, 31.1%) and private (983/1426, 68.9%) hospitals and randomized into the 1 of the 4 trial arms (control n=358, FFABC n=338, Milk Man n=397, and combination n=333). Of these, 76.6% (1092/1426) of fathers completed the baseline questionnaire, 86.8% (1238/1426) notified the project of the birth of their baby via SMS text survey, 58.6% (836/1426) completed the 6-week follow-up questionnaire, and 49.2% (702/1426) completed the 26-week follow-up questionnaire. Fathers recruited from private hospitals were significantly more likely to complete the baseline questionnaire than fathers recruited from public hospitals (808/983, 82.2%, vs 284/443, 64.1%; *P*<.001). Overall, 7.6% (108/1426) of recruited fathers provided no data and 43.1% (614/1426) provided complete data, with no discernible differences in level of participation in data collection surveys seen between the 4 intervention groups (Multimedia Appendix 1).

The average age of fathers who completed the baseline questionnaire was 33.6 (SD 5.2) years; the majority were born in Australia (724/1074, 67.4%) and had attended university (663/1072, 61.8%). There were no differences in the baseline characteristics between the 4 intervention groups (Table 1).

**Table 1:** Baseline characteristics of participating fathers by intervention group (n=1092).

Characteristic		Control (n=271)	FFABC^a^ (n=263)	Milk Man (n=299)	Combination (n=259)	Total	*P* value
**Age in years, mean (SD)**		33 (4.8)	34 (4.7)	34 (5.3)	34 (5.7)	33 (5.2)	.10
**Education, n (%)**		—^b^	—	—	—	—	.64
	High school/trade	109 (41.0)	99 (38.7)	106 (35.8)	95 (37.4)	409 (38.2)	
	Some/completed university	157 (59.0)	157 (61.3)	190 (64.2)	159 (62.6)	663 (61.8)	
**Place of birth, n (%)**		—	—	—	—	—	.93
	Australia/New Zealand	187 (70.0)	172 (67.2)	199 (67.2)	166 (65.1)	724 (67.4)	
	United Kingdom/Ireland	27 (10.1)	33 (12.9)	38 (12.8)	31 (12.2)	129 (12.0)	
	Africa/Middle East	14 (5.2)	12 (4.7)	20 (6.8)	19 (7.5)	65 (6.1)	
	Asia	23 (8.6)	22 (8.6)	21 (7.1)	18 (7.1)	84 (7.8)	
	Other	16 (6.0)	17 (6.6)	18 (6.1)	21 (8.2)	72 (6.7)	
**IRSAD^c^ deciles, n (%)**		—	—	—	—	—	.82
	1 and 2	8 (3.0)	7 (2.7)	7 (2.3)	6 (2.3)	28 (2.6)	
	3 and 4	7 (2.6)	8 (3.0)	10 (3.3)	9 (3.5)	34 (3.1)	
	5 and 6	62 (22.9)	44 (16.7)	59 (19.7)	58 (22.4)	223 (20.4)	
	7 and 8	53 (19.6)	67 (25.5)	75 (25.0)	65 (25.1)	260 (23.8)	
	9 and 10	141 (52.0)	137 (52.1)	149 (49.7)	121 (46.7)	548 (50.1)	
**Hospital, n (%)**		—	—	—	—	—	.85
	Public	110 (30.7)	100 (29.6)	124 (31.2)	109 (32.7)	443 (31.1)	
	Private	248 (69.3)	238 (70.4)	273 (68.8)	224 (67.3)	983 (68.9)	

^a^FFABC: father-focused antenatal breastfeeding class.

^b^Not applicable.

^c^IRSAD: Index of Relative Social Advantage and Disadvantage, where 1 = most disadvantaged and 10 = least disadvantaged.

### Intention to Treat Analysis

There were no significant differences between intervention arms in the proportion of infants being exclusively breastfed at 6 weeks and 26 weeks of age or in the proportion of infants receiving any breast milk at these ages (Multimedia Appendix 2). There were no significant differences between intervention arms in the risk of stopping exclusive breastfeeding or any breastfeeding before 26 weeks. Similarly, there were no significant differences between intervention arms in the risk of introducing formula or complementary foods before 26 weeks (Table 2). Also, there were no differences between intervention arms in the level of maternal breastfeeding confidence or postpartum partner support reported by mothers (Table 3).

**Table 2:** Comparison between control and intervention groups of risk of cessation of exclusive and any breastfeeding and introduction of formula and solids before 26 weeks: intention-to-treat analysis.

Intervention arm		Exclusive breastfeeding				Any breastfeeding					Introduction of formula					Introduction of complementary foods		
		HR^a^		95% CI		HR		95% CI		HR			95% CI		HR			95% CI
**Original^b^**																		
	Control	1.00	—^c^		1.00		—		1.00			—		1.00			—	
	FFABC^d^	1.09	0.91-1.32		1.01		0.67-1.51		1.19			0.90-1.56		1.08			0.86-1.35	
	Milk Man app	1.04	0.87-1.25		1.08		0.73-1.58		1.07			0.81-1.39		1.06			0.85-1.33	
	Combination	0.97	0.80-1.18		0.90		0.60-1.35		0.89			0.67-1.19		0.91			0.72-1.15	
**Pooled^e^**																		
	Control	1.00	—		1.00		—		1.00			—		1.00			—	
	FFABC	1.11	0.86-1.42		1.06		0.57-1.99		1.18			0.64-2.21		1.09			0.80-1.48	
	Milk Man app	1.04	0.81-1.35		1.13		0.59-2.18		1.13			0.62-2.06		1.13			0.81-1.58	
	Combination	0.98	0.73-1.31		0.89		0.47-1.70		0.90			0.48-1.68		1.02			0.75-1.38	

^a^HR: hazard ratio.

^b^Original analyses included those participants with complete data.

^c^Not applicable.

^d^FFABC: father-focused antenatal breastfeeding class.

^e^Pooled analyses that used the imputed datasets.

**Table 3:** Comparison of breastfeeding self-efficacy and postpartum partner support between control and intervention groups: intention-to-treat analysis.

Intervention arm				Mean		95% CI	β		SE		*P* value
**BSES-SF^a^**											
	**Original^b^**										
		Control	49.5		48.0-51.0		Ref	—^c^		—	
		FFABC^d^	48.7		47.1-50.3		–0.748	1.123		.51	
		Milk Man app	50.1		48.4-51.3		0.379	1.081		.73	
		Combination	49.5		48.5-51.6		0.589	1.111		.60	
	**Pooled^e^**										
		Control	47.4		45.0-49.7		Ref	—		—	
		FFABC	47.3		44.9-49.6		–0.112	1.677		.95	
		Milk Man app	48.3		46.1-50.5		0.919	1.731		.60	
		Combination	47.9		46.0-49.8		0.542	1.532		.72	
**PPSS^f^**											
	**Original**										
		Control	82.8		81.4-84.2		Ref	—		—	
		FFABC	82.5		81.0-83.9		–0.317	1.033		.76	
		Milk Man app	83.1		81.7-84.4		0.256	0.994		.80	
		Combination	81.2		79.8-82.7		1.595	1.026		.12	
	**Pooled**										
		Control	81.7		79.2-84.2		Ref	—		—	
		FFABC	81.0		78.1-83.9		–0.680	2.023		.74	
		Milk Man app	82.8		80.2-85.4		1.146	1.765		.52	
		Combination	78.7		75.3-82.0		–2.991	2.161		.17	

^a^BSES-SF: Breastfeeding Self-Efficacy Scale–Short Form, with scores ranging from 14 to 70 with higher scores indicating higher levels of breastfeeding self-confidence.

^b^Original analyses included those participants with complete data.

^c^Not applicable.

^d^FFABC: father-focused antenatal breastfeeding class.

^e^Pooled analyses that used the imputed datasets.

^f^PPSS: Postpartum Partner Support Scale, with scores ranging from 25 to 100 with higher scores indicating higher levels of postpartum partner support.

### Per Protocol Analysis

Overall, 85.1% (1214/1426) of fathers were eligible to be included in the per-protocol analysis. This included the entire control group (n=358); 87.9% (297/338) of the FFABC group, who had attended the class; 80.4% (319/397) of the Milk Man app group, who had downloaded the app; and 72.1% (240/333) of the combination group, who had attended the antenatal class and downloaded the Milk Man app. Significantly more of the participants recruited from private hospitals (871/983, 88.6%) were included in the per-protocol analysis than those recruited from the public hospitals (343/443, 77.4%; *P*<.001). Overall, there were no differences in the age, level of education, or social disadvantage of those who did or did not participate in the intervention per protocol. Within the individual intervention arms, participants recruited from public hospitals were significantly less likely to participate in any of the 3 interventions compared with those recruited from private hospitals. Younger fathers were less likely to participate in the FFABC or to download the Milk Man app, and fathers from the most disadvantaged group were less likely to participate in the FFABC (Multimedia Appendix 3).

Similar to the intention-to-treat analysis, the per-protocol analysis did not identify any significant differences between intervention arms for any of the primary or secondary outcome variables investigated (Multimedia Appendix 4).

### Milk Man Engagement Analysis

An engagement index for participants in the Milk Man and combination intervention arms was calculated using app analytics data and data from the 6-week follow-up questionnaire [49]. There were no differences in the engagement index scores between participants in the Milk Man and the combination intervention groups, and level of engagement was not associated with breastfeeding outcomes (data not presented) [49].

## Discussion

### Principal Findings

To our knowledge, PIFI is the largest breastfeeding intervention targeting fathers. We have previously reported on the process evaluation of the interventions and demonstrated that each interventions in terms of intent, content, and delivery was feasible, useful, and acceptable to fathers [34,39,50]. We were, however, unable to demonstrate impact of a face-to-face or mHealth intervention, either individually or in combination, on infant feeding outcomes, maternal breastfeeding self-efficacy, or level of postpartum partner support.

### Comparison With Prior Work

One of the interventions was a face-to-face antenatal breastfeeding class led by a trained peer facilitator. Breastfeeding peer support programs for fathers have previously been shown to be effective in increasing breastfeeding initiation rates and prolonging breastfeeding duration among socially disadvantaged couples [27-29]. Members of the research team had previously demonstrated in FIFI that a male-facilitated antenatal class of this type, supported by printed and promotional materials at weekly intervals for the first 6 weeks postpartum, resulted in a significantly larger proportion of infants being breastfed at 6 weeks compared with the usual care [25].

Building on the feedback from participants and lessons learned in FIFI, we refined and updated the content of the FFABC, and 117 FFABCs with an average size of 4 to 6 participants were delivered by a team of 11 trained peer facilitators [39]. A short process evaluation survey was completed by 98% of class attendees, and overall satisfaction with class format, facilitation, and content was high. Participants appreciated the validation of their role and valued the opportunity to interact with other fathers. Many fathers were not aware of the importance of or potential difficulties with breastfeeding and found the discussion around parenting and specific breastfeeding support strategies valuable [39].

We did not achieve the impact of FIFI with the FFABC in this study, which may be explained by differences in the participants of the 2 studies. Participants in FIFI, which was a smaller study (n=699), were all recruited from public hospitals and only 21% were tertiary educated. In contrast, the large target sample size required for PIFI, due to the 4-arm factorial design of the RCT, necessitated the recruitment of fathers from almost all maternity services across Perth, including private hospitals, which are responsible for approximately 50% of all births in Perth [37]. A disproportionate number of participants (983/1426) was recruited from private hospitals with just under one-third of participants being recruited from public hospitals. Additionally, half of the couples resided in the most socially advantaged areas of Perth. While initiation rates are high (>90%) among Australian women regardless of socioeconomic status [7], there is a persistent gap in the duration of exclusive and any breastfeeding between the most disadvantaged and least disadvantaged women in Australia [7,51]. Similarly, almost two-thirds of fathers and three-quarters of mothers in PIFI were tertiary educated. Maternal education has been consistently shown to be positively associated with successful breastfeeding outcomes [52,53].

There is evidence of a digital and health literacy divide, with both being directly associated with education and income [54-56]. This has important implications for digital health research projects such as PIFI, as individuals with lower health literacy may be less willing and able to participate in research that requires engagement with digital technology [54]. The characteristics of the PIFI sample indicate that we recruited a socially advantaged and highly educated sample that likely was highly digitally and health literate and as a consequence familiar with infant feeding recommendations and strongly motivated to breastfeed before entering the trial.

A key recommendation from the process evaluation of FIFI was that technology be employed in the form of internet websites and email contact to provide postnatal support for time-poor fathers [43]. FIFI was conducted between May 2008 and June 2009, and in the intervening period the technological landscape had changed, and smartphone apps increasingly were being developed and used to deliver mHealth interventions [30]. The decision was made, therefore, to develop a smartphone app for use in PIFI; the design, development, and formative evaluation of the Milk Man app has been described in detail previously [44].

The Milk Man app was downloaded by 8 of 10 participants who were randomized to either the Milk Man or combination group. As this was the first app of its kind designed especially for fathers, there is no other study to compare it with. However, an extensive process evaluation of the app was undertaken as part of the PIFI [50] using a comprehensive and customized evaluation framework, which in addition to determining the impact and efficacy of the app, also examined elements such as the robustness of the technology, the intervention principles and engagement strategies, and the interaction of the user with the technology [57]. The design and ease of use of the app rated highly, and overall, users’ opinions of the app were positive, with two-thirds indicating that they would recommend the app to other fathers [50].

The app included a customized app analytics framework that tracked how and when individual fathers were using the app over time. From approximately 32 weeks’ gestation to 6 weeks postpartum, there were more than 79,000 in-app user interactions, with app use being concentrated in the weeks around the birth of the baby. The conversation forum was the hub of app activity, with conversation starters prompting the reading of library articles (average of 11.5 per user) and all but one of the most accessed library articles and external organization links being associated with the conversation forum. Active engagement in the conversation forum was relatively high, with approximately one-third of fathers posting comments in the conversation forum 1126 times (average of 2.21 per user) and voting in polls 3096 times (average of 6 per user) [50]. This is higher than that reported in other studies [58,59], and it should be noted that lurkers (those who observe but don’t post) may experience benefit as well [58]. Qualitative data collected in the 6-week follow-up questionnaire from fathers randomized to either the Milk Man or combination group indicated that fathers used the online forum in a variety of ways to facilitate social support and share information and experiences with other fathers [34].

### Strengths and Limitations

Strengths of PIFI are that both interventions were designed with input from the end user. Another strength is that Milk Man app use was not prescribed, instead fathers were invited to use the app of their own volition, as they would in real life. As a result, there was wide variation in use patterns, which is likely to reflect real-life app engagement [50].

There are a number of limitations to this study, the first being that recruitment took longer than anticipated and for funding reasons was stopped prior to recruiting the target sample of 1600 couples. Although almost 90% of the target sample was recruited, attrition from the study was higher than the anticipated 25%, with less than half of recruited fathers providing complete baseline and follow-up data. As a result, the study was underpowered. While for convenience, follow-up questionnaires were administered online, they contained validated instruments designed to measure a variety of psychosocial factors associated with breastfeeding and parenting [36]. Therefore, questionnaires were relatively lengthy and time consuming to complete.

In this study, response rates for the short surveys delivered via SMS text were higher than that for the online surveys, with more than 8 of 10 fathers responding to the weekly SMS text surveys sent from 36 weeks’ gestation until the birth and inquiring about the arrival of their baby. Similarly, 8 of 10 and 7 of 10 mothers responded to the short infant feeding SMS text surveys administered at 12 weeks and 18 weeks, respectively. Frequent app-based breastfeeding data collected from mothers has been validated against other more labor-intensive methods such as self-administered questionnaires and health visitor reports and shown to reduce participant burden and provide reliable, more complete data [60]. Therefore, in the future in order to reduce respondent burden and attrition and gather more complete data, we recommend collecting minimal data related to feeding outcomes of interest via frequent but short surveys administered from within the app or via SMS text.

The focus on a family structure of male and female identifying partners was another limitation of this study. However, resources were not available to adapt the individual interventions for specific sexual and gender minority groups. As such, single parents and same-sex couples were excluded from the study. Further research to adapt the intervention for specific population groups is warranted.

The major limitation of the study, however, was that participants in this study, although randomly assigned to an intervention arm, were self-selected, and the resulting sample was not representative of the general population of expecting parents. Self-selection bias has been reported for other family-based studies involving fathers, with bias tending to be in the direction of overrepresenting those of higher educational attainment and those who are more invested in their fathering role [61]. Self-selection bias of this kind affected the generalizability of our findings, and had we recruited a more socioeconomically diverse sample of fathers, we may have seen an effect of the FFABC similar to that reported previously for FIFI [25] and other peer-facilitated face-to-face interventions involving socially disadvantaged fathers [27-29]. This self-selection bias would also have contributed to our inability to detect an impact of the Milk Man app on the primary breastfeeding outcomes or secondary outcomes, including postpartum partner support.

### Conclusions

This study did not demonstrate a measurable impact of either a peer-facilitated, face-to-face, father-focused breastfeeding class or a breastfeeding smartphone app developed specifically for fathers. Nevertheless, neither intervention was shown to be inferior to the standard care delivered in routine antenatal classes, and process evaluation indicates that both interventions were acceptable to, and valued by, participant fathers. Face-to-face interventions are costly and difficult to sustain, but digital technologies such as smartphone apps provide the opportunity to deliver cost effective, safe, and scalable breastfeeding interventions to geographically dispersed populations. The Milk Man app is an innovative and highly acceptable approach to engage with expecting and new fathers seeking information and support. The acceptability and effectiveness of the app and the impact of its individual app-based engagement strategies, warrant further investigation. Ideally, Milk Man should be tested under pragmatic conditions designed to reduce barriers for those Australians who are less digitally included. Better understanding of how those who are less digitally included engage with smartphone-based health information will be of wide public health interest.

## Appendix

Multimedia Appendix 1 Participation in data collection points by intervention arm.

Multimedia Appendix 2 Comparison of exclusive and any breastfeeding at 6 and 26 weeks between control and intervention groups: intention to treat analysis.

Multimedia Appendix 3 Percentage of participants completing the intervention per protocol by sociodemographic characteristics and intervention arm.

Multimedia Appendix 4 Results of per protocol analysis of primary and secondary outcomes.

Multimedia Appendix 5 CONSORT-eHEALTH checklist (V 1.6.1).

## Abbreviations

BSES-SF: Breastfeeding Self-Efficacy Scale–Short Form
FFABC: father-focused antenatal breastfeeding class
FIFI: Father Infant Feeding Initiative
mHealth: mobile health
PIFI: Parent Infant Feeding Initiative
PPSS: Postpartum Partner Support Scale
RCT: randomized controlled trial
Reach HPI: Reach Health Promotion Innovations
SMS: short message service
