# Supplementary material for: Impact of a Face-To-Face Versus Smartphone App Versus Combined Breastfeeding Intervention Targeting Fathers: Randomized Controlled Trial
Source: JMIR Pediatr Parent. 2021 Apr 12;4(2):e24579. doi: 10.2196/24579 (PMC8076985; doi:10.2196/24579)
Supplement: Multimedia Appendix 1 [file pediatrics_v4i2e24579_app1.docx]

**Multimedia Appendix 1:** Participation in data collection points by intervention arm

| Characteristics | Total  n (%) | Control  n (%) | FFABC^a^  n (%) | Milk Man  n (%) | Combination  n (%) |
| --- | --- | --- | --- | --- | --- |
| Recruited/ randomized | 1426  (100.0) | 358  (25.1) | 338  (23.7) | 397  (27.8) | 333  (23.4) |
| Completed baseline survey | 1092  (76.6) | 271  (75.7) | 263  (77.8) | 299  (75.3) | 259  (77.8) |
| Notified birth of baby  (SMS) | 1238  (86.8) | 313  (87.4) | 286  (84.6) | 345  (86.9) | 295  (88.3) |
| Completed 6 weeks survey | 836  (58.6) | 215  (60.1) | 203  (60.1) | 224  (56.4) | 194  (58.3) |
| Completed 26 weeks survey | 702  (49.2) | 184  (51.4) | 177  (52.4) | 184  (46.3) | 157  (47.1) |
| No data provided | 108 (7.6) | 29  (8.1) | 27  (8.0) | 30  (7.6) | 22  (6.6) |
| Completed all data components | 614  (43.1) | 161  (45.0) | 163  (48.2) | 152  (38.3) | 138  (41.4) |
| Eligible for per protocol analysis^a^ | 1214  (84.8) | 358  (100) | 297  (87.9) | 319  (80.4) | 240  (72.1) |

^a^FFABC - attended class, Milk Man – downloaded app, Combination - attended class and downloaded
