# Supplementary material for: Impact of a Face-To-Face Versus Smartphone App Versus Combined Breastfeeding Intervention Targeting Fathers: Randomized Controlled Trial
Source: JMIR Pediatr Parent. 2021 Apr 12;4(2):e24579. doi: 10.2196/24579 (PMC8076985; doi:10.2196/24579)
Supplement: Multimedia Appendix 2 [file pediatrics_v4i2e24579_app2.docx]

**Multimedia Appendix 2.** Comparison of exclusive and any breastfeeding at 6 and 26 weeks between control and intervention groups: intention to treat analysis

| Intervention arm | Exclusive BF  6 weeks | | | Any BF  6 weeks | | | Exclusive BF  26 weeks | | | Any BF  26 Weeks | | |
| --- | --- | --- | --- | --- | --- | --- | --- | --- | --- | --- | --- | --- |
|  | (%) | OR | 95% CI | (%) | OR | 95% CI | (%) | OR | 95%CI | (%) | OR | 95% CI |
| ***Original^a^*** |  |  |  |  |  |  |  |  |  |  |  |  |
| Control | (71) | 1.00 |  | (94) | 1.00 |  | (5) | 1.00 |  | (80) | 1.00 |  |
| FFABC | (67) | 0.82 | 0.56-1.19 | (93) | 0.88 | 0.45-1.72 | (3) | 0.48 | 0.18-1.26 | (72) | 0.81 | 0.52-1.24 |
| Milk Man App | (69) | 0.91 | 0.63-1.30 | (93) | 0.85 | 0.44-1.61 | (4) | 0.82 | 0.37-1.80 | (78) | 0.90 | 0.59-1.37 |
| Combination | (74) | 1.13 | 0.76-1.66 | (95) | 1.38 | 0.65-2.92 | (5) | 0.97 | 0.44-2.14 | (82) | 1.16 | 0.74-1.82 |
| ***Pooled^b^*** |  |  |  |  |  |  |  |  |  |  |  |  |
| Control | (67) | 1.00 |  | (84) | 1.00 |  | (18) | 1.00 |  | (72) | 1.00 |  |
| FFABC | (63) | 0.83 | 0.32-2.14 | (82) | 0.86 | 0.32-2.27 | (18) | 1.03 | 0.35-3.00 | (67) | 0.80 | 0.31-2.02 |
| Milk Man App | (65) | 0.92 | 0.36-2.32 | (82) | 0.86 | 0.29-2.55 | (19) | 1.13 | 0.40-3.21 | (68) | 0.82 | 0.33-2.08 |
| Combination | (68) | 1.06 | 0.40-2.82 | (85) | 1.11 | 0.39-3.18 | (19) | 1.06 | 0.35-3.22 | (72) | 1.01 | 0.39-2.62 |

^a^ the original analyses included those participants with complete data

^b^ the pooled analyses which used the imputed datasets
