# Supplementary material for: Impact of a Face-To-Face Versus Smartphone App Versus Combined Breastfeeding Intervention Targeting Fathers: Randomized Controlled Trial
Source: JMIR Pediatr Parent. 2021 Apr 12;4(2):e24579. doi: 10.2196/24579 (PMC8076985; doi:10.2196/24579)
Supplement: Multimedia Appendix 3 [file pediatrics_v4i2e24579_app3.docx]

**Multimedia Appendix 3:** Percentage of participants completing the intervention per protocol by socio-demographic characteristics and intervention arm

|  | Control  *(n= 358)* | FFABC^a^  *(n 297)* | Milk Man  *(n 319)* | Combination  *(n 240)* | Total  *(n=1214)* |
| --- | --- | --- | --- | --- | --- |
| Age group (years) |  | p=0.008 | p=0.018 | p=0.539 | p=0.256 |
| < 30 | 100.0 | 81.0 | 84.3 | 80.4 | 87.6 |
| 30-34 | 100.0 | 95.7 | 85.7 | 84.3 | 91.3 |
| ≥ 35 | 100.0 | 93.3 | 96.1 | 78.4 | 91.5 |
| Education |  | p= 0.074 | p=0.365 | p=0.347 | p=0.086 |
| High school/trade | 100.0 | 88.9 | 87.7 | 78.9 | 89.2 |
| Some/ completed university | 100.0 | 94.9 | 91.1 | 83.6 | 92.3 |
| Place of birth |  | p=0.439 | p=0.286 | p=0.150 | p=0.047 |
| Australia/New Zealand | 100.0 | 93.0 | 90.5 | 83.1 | 91.5 |
| United Kingdom/ Ireland | 100.0 | 97.0 | 92.1 | 74.2 | 90.7 |
| Africa/ Middle East | 100.0 | 83.3 | 90.0 | 78.9 | 87.7 |
| Asia | 100.0 | 86.4 | 76.2 | 66.7 | 83.3 |
| Other | 100.0 | 94.1 | 94.8 | 95.2 | 95.8 |
| IRSAD^b^ deciles, |  | p=0.008 | p=0.253 | p=0.197 | p=0.320 |
| 1 and 2 | 100.0 | 57.1 | 100 | 66.7 | 82.1 |
| 3 and 4 | 100.0 | 100.0 | 70.0 | 100.0 | 91.2 |
| 5 and 6 | 100.0 | 90.9 | 86.4 | 75.9 | 88.3 |
| 7 and 8 | 100.0 | 92.5 | 89.3 | 87.7 | 91.9 |
| 9 and 10 | 100.0 | 94.2 | 90.6 | 79.3 | 91.4 |
| Hospital  Public  Private | 100.0  100.0 | p=0.001  79.0  91.6 | p=0.002  71.0  84.6 | p=0.001  60.6  77.7 | p<0.001  77.4  88.6 |

^a^ FFABC Father-focused antenatal breastfeeding class

^b^ IRSAD Index of Relative Social Advantage and Disadvantage where 1 = most disadvantaged and 10 = least disadvantaged
