# Supplementary material for: Impact of a Face-To-Face Versus Smartphone App Versus Combined Breastfeeding Intervention Targeting Fathers: Randomized Controlled Trial
Source: JMIR Pediatr Parent. 2021 Apr 12;4(2):e24579. doi: 10.2196/24579 (PMC8076985; doi:10.2196/24579)
Supplement: Multimedia Appendix 4 [file pediatrics_v4i2e24579_app4.docx]

**Multimedia Appendix 4** Results of per protocol analysis of primary and secondary outcomes

**Table 1.** Comparison of exclusive and any breastfeeding at 6 and 26 weeks between control and intervention groups: per protocol analysis

| Intervention arm | Exclusive BF  6 weeks | | | Any BF  6 weeks | | | Exclusive BF  26 weeks | | | Any BF  26 Weeks | | |
| --- | --- | --- | --- | --- | --- | --- | --- | --- | --- | --- | --- | --- |
|  | (%) | OR | 95% CI | (%) | OR | 95% CI | (%) | OR | 95%CI | (%) | OR | 95% CI |
| ***Original^a^*** |  |  |  |  |  |  |  |  |  |  |  |  |
| Control | (71) | REF |  | (94) | REF |  | (5) | REF |  | (80) | REF |  |
| FFABC | (67) | 0.83 | 0.57-0.22 | (93) | 0.85 | 0.43-1.69 | (2) | 0.42 | 0.15-1.20 | (76) | 0.79 | 0.51=1.23 |
| Milk Man App | (70) | 0.92 | 0.53-1.33 | (93) | 0.96 | 0.49-1.88 | (4) | 0.72 | 0.32-1.66 | (78) | 0.90 | 0.59-1.39 |
| Combination | (74) | 1.11 | 0.74-1.68 | (97) | 1.90 | 0.78-4.63 | (5) | 0.89 | 0.38-2.11 | (83) | 1.21 | 0.74-1.97 |
| ***Pooled^b^*** |  |  |  |  |  |  |  |  |  |  |  |  |
| Control | (67) | REF |  | (84) | REF |  | (18) | REF |  | (72) | REF |  |
| FFABC | (64) | 0.85 | 0.35-2.09 | (83) | 0.93 | 0.36-2.43 | (16) | 0.87 | 0.30-2.58 | (68) | 0.83 | 0.34-2.04 |
| Milk Man App | (67) | 0.98 | 0.45-2.17 | (88) | 1.32 | 0.46-3.77 | (14) | 0.75 | 0.28-1.98 | (71) | 0.98 | 0.46-2.08 |
| Combination | (70) | 1.11 | 0.47-2.64 | (90) | 1.65 | 0.53-5.15 | (15) | 0.82 | 0.28-2.40 | (75) | 1.20 | 0.51-2.84 |

^a^ the original analyses included those participants with complete data

^b^ the pooled analyses which used the imputed datasets

**Table 2.** Comparison between control and intervention groups of risk of cessation of exclusive and any breastfeeding, and introduction of formula and solids before 26 weeks: per protocol analysis

| Intervention arm | Exclusive Breastfeeding | | Any breastfeeding | | Introduction of formula | | Introduction of complementary foods | | |
| --- | --- | --- | --- | --- | --- | --- | --- | --- | --- |
|  | HR | 95% CI | HR | 95% CI | HR | 95%CI | | HR | 95% CI |
| ***Original^a^*** |  |  |  |  |  |  | |  |  |
| Control | REF |  | REF |  | REF |  | | REF |  |
| FFABC | 1.09 | 0.90-1.32 | 1.03 | 0.68-1.56 | 1.16 | 0.88-1.54 | | 1.09 | 0.87-1.38 |
| Milk Man App | 1.07 | 0.88-1.28 | 1.03 | 0.69-1.53 | 1.09 | 0.83-1.43 | | 1.05 | 0.84-1.32 |
| Combination | 0.96 | 0.79-1.18 | 0.87 | 0.56-1.36 | 0.93 | 0.69-1.26 | | 0.92 | 0.72-1.18 |
| ***Pooled^b^*** |  |  |  |  |  |  | |  |  |
| Control | REF |  | REF |  | REF |  | | REF |  |
| FFABC | 1.10 | 0.85-1.41 | 1.03 | 0.56-1.90 | 1.15 | 0.64-2.09 | | 1.13 | 0.84-1.52 |
| Milk Man App | 1.06 | 0.84-1.34 | 0.91 | 0.52-1.60 | 1.12 | 0.69-1.81 | | 1.12 | 0.83-1.51 |
| Combination | 0.97 | 0.74-1.27 | 0.77 | 0.43-1.40 | 0.91 | 0.53-1.58 | | 1.02 | 0.77-1.36 |

^a^ the original analyses included those participants with complete data

^b^ the pooled analyses which used the imputed datasets

**Table 3:** Comparison of breastfeeding self-efficacy and postpartum partner support between control and intervention groups: per protocol analysis

| Intervention arm | Mean | 95% CI | β | SE | p |
| --- | --- | --- | --- | --- | --- |
| **BSES-SF** |  |  |  |  |  |
| ***Original^a^*** |  |  |  |  |  |
| Control | 49.5 | 47.9-51.0 | REF |  |  |
| FFABC | 48.7 | 48.2-51.3 | -0.807 | 1.15 | 0.483 |
| Milk Man App | 49.8 | 47.6-51.0 | 0.331 | 1.101 | 0.763 |
| Combination | 49.3 | 47.9-51.0 | -0.173 | 1.175 | 0.883 |
| ***Pooled^b^*** |  |  |  |  |  |
| Control | 47.4 | 45.0-49.7 | REF |  |  |
| FFABC | 47.0 | 44.7-49.3 | -0.358 | 1.636 | 0.827 |
| Milk Man App | 48.8 | 47.1-50.6 | 1.440 | 1.511 | 0.342 |
| Combination | 47.6 | 45.8-49.3 | 0.205 | 1.496 | 0.891 |
|  |  |  |  |  |  |
| **PPSS** |  |  |  |  |  |
| ***Original*** |  |  |  |  |  |
| Control | 82.8 | 81.4-84.2 | Ref |  |  |
| FFABC | 82.4 | 80.9-83.9 | -0.362 | 1.046 | 0.730 |
| Milk Man App | 83.0 | 81.6-84.4 | 0.201 | 1.002 | 0.841 |
| Combination | 81.3 | 79.7-82.8 | -1.527 | 1.076 | 0.157 |
| ***Pooled*** |  |  |  |  |  |
| Control | 81.7 | 79.2-84.1 | REF |  |  |
| FFABC | 81.0 | 78.4-83.7 | -0.643 | 1.926 | 0.739 |
| Milk Man App | 82.6 | 80.7-84.5 | 0.942 | 1.576 | 0.551 |
| Combination | 79.4 | 76.8-82.0 | -2.257 | 1.844 | 0.223 |

^a^ the original analyses included those participants with complete data

^b^ the pooled analyses which used the imputed datasets

BSES-SF: Breastfeeding self-efficacy scale- short form, scores range from 14 to 70 with higher scores indicating higher levels of breastfeeding self-confidence

PPSS: Postpartum partner support scale, scores range from 25-100, with higher scores indicating higher levels of postpartum partner support
